# Validity of Endoscopic Submucosal Dissection for Gastric Cancer Diagnosed as Differentiated Adenocarcinoma Before Treatment Regardless of Lesion Size

**DOI:** 10.5152/tjg.2023.22611

**Published:** 2023-11-01

**Authors:** Kosuke Nomura, Shu Hoteya, Yorinari Ochiai, Takayuki Okamura, Yugo Suzuki, Junnosuke Hayasaka, Yutaka Mitsunaga, Masami Tanaka, Kazuhiro Fuchinoue, Hiroyuki Odagiri, Satoshi Yamashita, Akira Matsui, Daisuke Kikuchi

**Affiliations:** Department of Gastroenterology, Toranomon Hospital, Tokyo, Japan

**Keywords:** Endoscopic submucosal dissection, early gastric cancer, superficial spreading type

## Abstract

**Background/Aims:**

We investigated the validity and safety of endoscopic submucosal dissection for gastric tumors by examining short- and long-term outcomes by tumor diameter.

**Materials and Methods:**

Endoscopic submucosal dissection for gastric tumor was performed on 4259 lesions at our hospital between January 2005 and June 2021. [Study 1] Patients were divided into 5 tumor diameter groups: 3751 lesions, ≤30 mm; 366 lesions, 31-50 mm; 106 lesions, 51-75 mm; 24 lesions, 76-100 mm; and 12 lesions, ≥101 mm. Short-term gastric endoscopic submucosal dissection outcomes were investigated. [Study 2] Long-term outcomes (delayed gastric emptying and prognosis) were investigated in 508 cases with tumor diameter ≥31 mm.

**Results:**

[Study 1] Perforation rate (%) was 1.2, 3.6, 3.8, 12.5, and 25.0 for lesions with tumor diameter ≤30 mm, in the range 31-50 mm, 51-75 mm, and 76-100 mm, and ≥101 mm, respectively. Postoperative bleeding rate (%) was 4.8, 9.0, 6.6, 20.8, and 33.3, respectively, R0 resection rate (%) was 96.8, 90.2, 89.6, 70.8, and 66.6, respectively, and curative resection rate (%) was 92.0, 61.2, 63.2, 45.8, and 8.3, respectively. [Study 2] There were 7 cases of delayed gastric emptying after wide resection, with 3 patients requiring balloon dilatation, 1 of whom subsequently underwent distal gastrectomy. Among 205 cases of noncurative resection, 110 underwent additional surgery, residual cancer was present in 11 cases, and lymph node metastasis was observed in 7 cases (1 patient died of disease). To date, 1 of the 95 patients being followed up has died of disease (mean follow-up: 2042 days).

**Conclusion:**

Even with a tumor diameter ≥31 mm, curative resection was achieved in about 60% of cases in which intramucosal lesions were considered possible preoperatively, but the rate was low at 8.3% for tumor diameter ≥101 mm. Long-term outcomes appear favorable, with only 0.4% of the patients dying of disease but delayed gastric emptying observed in 1.7% of cases.

**Main Points**

- Endoscopic submucosal dissection for tumor diameters greater than 31 mm showed that the R0 resection rate and curative resection rate decreased with increasing tumor diameter, while procedure duration and complications increased.
- Curative resection was achieved in approximately 60% of cases, even for tumor diameters larger than 31 mm. However, for lesions larger than 100 mm, the curative resection rate was less than 10%.
- Long-term results were relatively good, with only 0.4% of patients dying of disease, but delayed gastric emptying occurred in 1.7% of cases.

## Introduction

The advent of endoscopic submucosal dissection (ESD) for the treatment of early gastric cancer has enabled en bloc resection of lesions that are large or accompanied by ulceration (UL)/scarring. Endoscopic submucosal dissection also facilitates accurate histopathological diagnosis.^1^ Now widely disseminated, ESD is considered a standard treatment. It allows patients to avoid surgical resection while ensuring a favorable prognosis, and the indications continue to expand.

Differentiated intramucosal carcinoma without UL is a diagnosis of curative resection regardless of the lesion size.^2^ On the other hand, for cancer with UL or submucosal (SM) invasion, the decision for noncurative resection is made if the tumor diameter is 31 mm or larger. Accurate diagnosis by endoscopy or endoscopic ultrasonography is not easy,^3-5^ and diagnostic treatment is often performed for lesions larger than 31 mm. To date, there has been no report of a focused study of lesions larger than 31 mm. Therefore, in patients with endoscopic intramucosal lesions, we investigated the validity and safety of ESD for gastric tumors at our hospital by investigating the short- and long-term outcomes according to tumor diameter (TD) in order to determine the probability of curative resection at larger TD and to determine whether there is an increase in the incidence of procedural complications.

## Materials and Methods

In this study, ESD for gastric tumor was performed on 4259 lesions at our hospital between January 2005 and June 2021.

[Study 1] Patients were divided into 5 groups: 3751 lesions with a TD ≤30 mm; 366 lesions with a TD in the range 31-50 mm; 106 lesions with a TD in the range 51-75 mm; 24 lesions with a TD in the range 76-100 mm; and 12 lesions with a TD ≥101 mm. We then investigated the short-term gastric ESD outcomes. Clinicopathological characteristics, tumor size (mm), procedure duration (minutes), rate of general anesthesia, complete (R0) resection rate, curative resection rate, and postoperative bleeding and perforation were evaluated. [Study 2] We investigated the long-term outcomes [delayed gastric emptying (DGE) and overall survival rates] in 508 cases (498 patients) with TD ≥31 mm.

All patients provided written informed consent to undergo the proposed procedure. The study was approved by the Institutional Review Board of Toranomon Hospital (approval no.  1905).

The primary outcome of this study was rates of curative resection. Secondary outcomes of this study were clinicopathological characteristics, rate of complete resection, postoperative bleeding, perforation, and DGE.

### Indications

Indications for treatment of the lesions were determined based on endoscopic and biopsy findings. Indications for ESD were (i) differentiated-type intramucosal cancer without UL, irrespective of tumor size; (ii) differentiated-type intramucosal cancer with UL and TD <30 mm; or (iii) adenoma with malignant potential. Endoscopic ultrasonography was performed using a miniature probe (UM2R or UM3R; Olympus Medical Systems Corp., Tokyo, Japan) when SM invasion or UL was suspected. In patients who were considered to have possible SM invasion, computed tomography (CT) was performed to exclude any regional lymph node metastasis or distant metastasis.

### Endoscopic Submucosal Dissection Procedure

**Figure 1.**
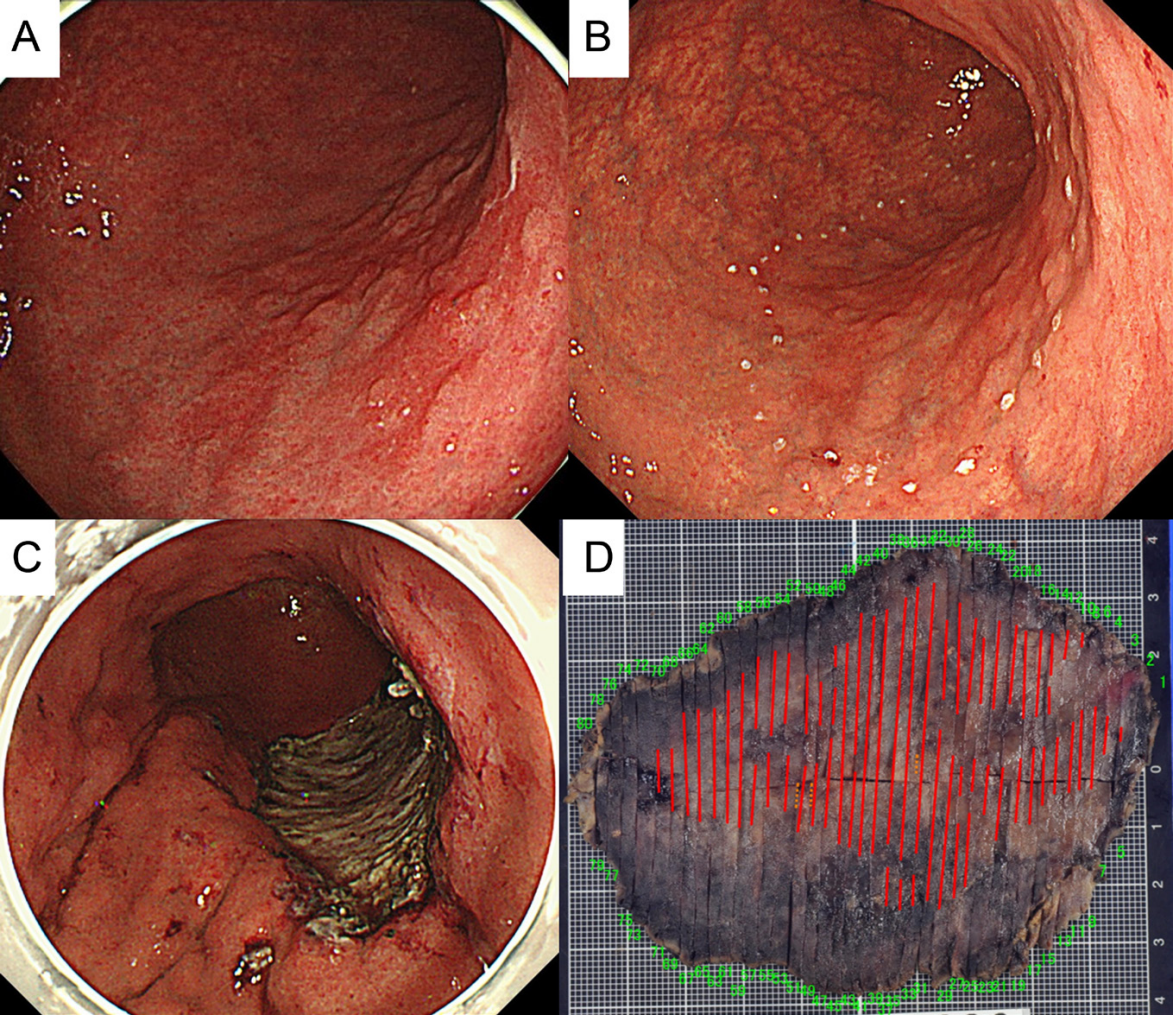
The endoscopic submucosal dissection procedure. (A) Endoscopic image of a lesion before endoscopic submucosal dissection (ESD). The lesion was mainly located on the posterior wall from the middle to the lower body. (B) Preoperative marking image. (C) Ulceration after ESD. (D) Histological mapping of a specimen after ESD. Pathology results: 80 × 55 mm, 0-IIc, tub1, M, UL0, ly0, v0, pHMO, pVM0. Curative resection. Red line indicates a mucosal lesion.

The ESD procedure was performed with a dual knife (KD-650L or KD-655L; Olympus Medical Science, Tokyo, Japan) through a 2-channel scope equipped with multibending and water jet functions (GIF-2TQ260 M; Olympus Medical Science) or a single-channel endoscope (GIF-Q260J; GIF-H290T; Olympus Medical Science), as previously reported.^6-8^ The electrosurgical unit used was a VIO300D or VIO3 (ERBE, Tübingen, Germany). In brief, a soft transparent hood (D-201-13404; Olympus Optical, Tokyo, Japan) was attached to the tip of the endoscope to obtain good, direct endoscopic views of the SM layer. Some marking dots were placed on the normal mucosa approximately 5 mm from the tumor margin to provide safety margins. After SM injection of glycerol (10% glycerol and 5% fructose; Chugai Pharmaceutical, Tokyo, Japan) with a small amount of indigo carmine and 0.1% epinephrine, a mucosal incision was made outside the marking dots. Hyaluronic acid solution was added to the injection solution when mucosal elevation was insufficient due to UL of the lesion or massive fibrosis of the SM layer.^9^ After mucosal incision, the SM layer was dissected directly to obtain an intact specimen, and complete en bloc resection was performed (Figure 1A–D). Hemostatic forceps (HDB2422 W; Pentax, Tokyo, Japan) in soft coagulation mode were used to control bleeding during the procedure. In all cases, prophylactic coagulation of visible vessels on the mucosal defect was performed immediately after ESD with hemostatic forceps or with hemostatic clips (EZ Clips; Olympus Medical Systems or SureClip; Micro-Tech). Endoscopic submucosal dissection was usually performed in conscious patients sedated with a mixture of diazepam (5-10 mg) and pethidine hydrochloride (35-70 mg). However, if the procedure was expected to take longer than 2 hours, general anesthesia was administered. The procedure time was calculated from the start of marking around the lesion to the end of resection.

### Histological Assessment

The resected specimen was cut into 2-mm slices after fixation in 10% formalin. Histological type, depth of invasion, lateral or vertical margins, and lymphovascular invasion were evaluated in each slice according to the Japanese Classification of Gastric Carcinoma.^10^

R0 resection was defined as resection in 1 piece with tumor-free margins. However, this definition did not include the depth of invasion, lymphovascular infiltration, or the type of adenocarcinoma. Curative resection was defined as a resected specimen meeting the requirements for R0 resection, without invasion into a lymph duct or venous duct, and meeting 1 of the following 4 Japanese Gastric Cancer Association specifications^2^: (i) differentiated-type mucosal cancer without UL; (ii) differentiated-type mucosal cancer with UL and TD ≤30  mm; (iii) differentiated-type minute SM cancer (SM1) and TD ≤30  mm; (iv) undifferentiated-type mucosal cancer without UL and TD ≤20  mm.

Tumor histopathology was evaluated with ESD specimens, and mixed carcinoma was defined as the presence of both differentiated and undifferentiated histologic types. Tumor diameter was also evaluated in ESD specimens.

### Follow-up After Endoscopic Submucosal Dissection

Patients treated with curative ESD were followed-up using endoscopy examination every 6-12 months. For patients treated with noncurative ESD, patients at possible risk of lymph node metastasis were recommended to have additional surgery with lymph node dissection. In addition, abdominal CT or abdominal ultrasonography and endoscopy were performed every 6-12 months in noncurative ESD patients undergoing additional gastrectomy. Patients with noncurative ESD who did not undergo surgical resection were followed-up endoscopically with additional abdominal CT or ultrasonography every 6 months for at least 3 years, with an annual follow-up thereafter.

### Complications

Postoperative bleeding was defined as bleeding requiring emergency endoscopy or transfusion or as a decrease in hemoglobin level of >2 g/dL following ESD. Perforation was diagnosed endoscopically or based on the presence of free air on plain abdominal x-ray or CT after ESD.

### Statistical Analysis

Data are presented as the mean ± SD. Statistical analysis was performed using the chi-squared test, Mann–Whitney *U*-test, and 1-way analysis of variance. All statistical analyses were performed using Stata version 14 (StataCorp, College Station, Tex, USA). *P* <.05 was considered statistically significant.

## Results

### Study 1

Table 1 shows the clinicopathological characteristics of the patients. All patients were in their early 70s, and there was a greater percentage of women in the TD 76-100 mm group. The mean procedure duration was 70.3, 121.2, 166.3, 246.0, and 290.9 minutes in the TD ≤30 mm, TD 31-50 mm, TD 51-75 mm, TD 76-100 mm, and TD ≥101 mm groups, respectively. These results showed that the procedure duration increased significantly with increasing lesion size. The frequency of general anesthesia and the percentage of tumors with mixed histology also increased significantly with increasing TD.

R0 resection (%) was achieved in 96.8%, 90.2%, 89.6%, 70.8%, and 66.6% of patients, with curative resection rates (%) of 92.0%, 61.2%, 63.2%, 45.8%, and 8.3% in the TD ≤30 mm, TD 31-50 mm, TD 51-75 mm, TD 76-100 mm, and TD ≥101 mm groups, respectively. Both of these results show that the curative resection rate decreased with increasing TD. The reasons for noncurative resection were SM invasion in 119 patients (58.0%), UL in 48 patients (23.4%), significantly poor differentiation in 25 patients (12.2%), a positive lateral margin in 34 patients (16.6%), and a positive vertical margin in 29 patients (14.1%) In terms of procedural complications, perforation (%) occurred in 1.2%, 3.6%, 3.8%, 12.5%, and 25.0% of patients and postoperative bleeding occurred in 4.8%, 9.0%, 6.6%, 20.8%, and 33.3%, respectively (Table 2). Therefore, complications also increased significantly with increasing TD. Although we cannot say that the rate of procedural complications was low by any means, no serious procedural complications were observed.

### Study 2

Except for 110 patients who underwent additional surgery, late procedural complications included 7 cases of DGE after wide resection (Table 3). The site involved was the antrum in 5 patients and the cardia in 2 patients. Six of these 7 patients had a mucosal defect around three-quarters or more of the circumference. There were 4 cases of DGE associated with gastric deformation and decreased peristalsis but without severe stenosis through which the scope could not pass; all of these cases improved after dietary restriction. Balloon dilatation was required to overcome stenosis in 3 cases. In 1 of those cases, there was no improvement despite a total of 8 balloon dilatation procedures; so we ultimately performed pyloric resection.

Among the 498 patients with early gastric cancer with TD ≥31 mm, curative resection was performed in 298 patients (303 lesions) and noncurative resection was performed in 200 patients (205 lesions). Among the cases of noncurative resection, 110 cases underwent additional surgical intervention, residual cancer was present in 11 cases, and lymph node metastasis was observed in 7 cases (1 patient died of disease). To date, only 1 of the 95 patients being followed up has died of disease (mean follow-up duration: 2042 days). No recurrence has been observed in the 298 patients who underwent curative resection.

## Discussion

Gastric ESD takes longer than endoscopic mucosal resection, but it has come to be widely accepted because it allows for en bloc resection of lesions that are large or accompanied by an ulcer scar.^11^ Endoscopic submucosal dissection has been covered by national health insurance in Japan since April 2006 and is now widely performed as standard treatment for early gastric cancer. In 2000, Gotoda et al^12^ demonstrated that the risk of lymph node metastasis from UL0 differentiated-type intramucosal carcinoma was extremely low, irrespective of the lesion size. Endoscopic submucosal dissection is now being performed for an increasing number of indications, including the aforementioned, based on preoperative diagnosis. Long-term outcomes of the Japan Clinical Oncology Group 0607 study were published in 2018, and the Japanese Gastric Cancer Treatment Guidelines^2^ were revised. Since then, such lesions such as the aforementioned have been included in the absolute indications. At present, the eCura scoring system is used to evaluate the degree of curative resection after endoscopic intervention for gastric cancer.^2^ A case of differentiated-type intramucosal early gastric cancer without ulceration (UL0) would be classified as eCura A, even if TD exceeds 31 mm. However, any SM invasion or UL in the lesion would lead to a diagnosis of eCura C2 and would require additional surgical resection.

The present study was limited to cases with possible intramucosal lesions suspected preoperatively, but curative resection was achieved in approximately 60% of cases, even if TD exceeded 31 mm. Tumor diameter was ≥76 mm in approximately 50% of cases, which was high. When we consider the benefit of gastric preservation, we feel that this is an extremely useful form of treatment. However, the rate of curative resection was less than 10% for lesions exceeding 101 mm in size, and appropriate treatment needs to be selected after making a holistic determination of the patient’s general condition.

The R0 resection rate decreased with increasing TD in our study. When TD was large, the frequency of damage to the specimen tended to increase and the vertical margin tended to be positive with a higher rate of SM invasion. Imagawa et al^13^ reported that the rate of en bloc resection decreased significantly when the lesion size exceeded 20 mm. The curative resection rate also decreased with increasing TD. Submucosal invasion was present in 20%-30% of cases with TD 31-50 mm or 51-75 mm, compared with approximately 40% for TD 76-100 mm and approximately 60% for TD ≥101 mm. Even though no sites had conspicuous superficial irregularity during endoscopy, SM invasion was found when we attempted to resect larger lesions in practice. Ulceration was observed in approximately 15% of lesions with TD 31-50 mm, 51-75 mm, or 76-100 mm and in 30% of lesions with TD ≥101 mm. In this study, we did not observe any noteworthy trends in TD or UL.

It has been reported that the percentage of undifferentiated tumors increases with increasing TD. Reports suggest that this may be due to an increase in undifferentiated components as invasion by differentiated cancer continues, with undifferentiated components ultimately becoming predominant as time progresses.^14,15^ There are also reports of a tendency toward increased mixed histological cancers with increasing TD,^16^ which is consistent with our present findings. However, we only performed ESD in cases diagnosed with differentiated-type cancer based on preoperative biopsy, and so the rate of patients with significantly poor differentiation in histology after ESD was quite low.

In this study, the procedure duration and procedural complications increased with increasing TD. Longer procedures are a natural consequence of a wider dissection area associated with increased TD and this in turn leads to an increased risk of bleeding. In terms of procedural complications, there were some cases of perforation and postoperative bleeding associated with increased TD, but no serious procedural complications were observed, and the procedure could be performed safely. Minimizing the burden on the patient and the endoscopist is one way of ensuring safety during ESD for large lesions. The use of general anesthesia during long ESD procedures not only ensures patient safety but also allows the endoscopist to focus on the procedure by delegating anesthetic management. It is therefore mutually beneficial.

Late procedural complications included stenosis due to cicatricial contraction of a post-ESD ulcer and, although rare, DGE due to gastric deformation and decreased peristalsis. Larger TD may lead to problems in the cardia and pyloric antrum in particular, where resection of three-quarters or more of the circumference has been reported as a common risk factor for postoperative stenosis.^17,18^ During the present study, there were 3 cases that required balloon dilatation for stenosis after wide resection. One patient showed no improvement and required distal gastrectomy. In 4 cases, we did not observe stenosis but did observe DGE associated with deformation and decreased peristalsis. All of these cases required long-term dietary restriction but improved with conservative treatment.

No recurrence was observed in the patients who underwent curative resection. Although recurrence and death from disease occurred in 2 patients (1.0%) in the patients who underwent noncurative resection, overall the long-term outcome was favorable.

The limitations of the study are its retrospective design, its single-center setting in Japan, and the relatively small sample size. Despite these limitations, we believe this study makes a meaningful contribution because it is the first report on gastric ESD to focus on TD.

## Conclusion

In cases where tumor size exceeded 31 mm but intramucosal lesions were suspected preoperatively, curative resection was possible in about 60% of cases. However, the rate of curative resection was less than 10% for lesions exceeding 100 mm in size, and appropriate treatment needs to be selected after making a holistic determination of the patient’s general condition. The long-term outcomes appear favorable, with only 0.4% of our patients dying of disease but DGE occurring in 1.7% of cases.

## Figures and Tables

**Table 1.** Clinicopathological Features of Gastric ESD by Tumor Length

Tumor diameter (mm)	<30	31-50	51-75	76-100	>101	*P*
n	3751	366	106	24	12	
Age (years), mean ± SD	70.3 ± 9.6	70.6 ± 9.2	72.3 ± 10.9	74.2 ± 10.9	73.8 ± 4.5	.78
Sex (male), % (n)	78.0 (2924)	80.6 (295)	75.5 (80)	45.8 (11)	66.7 (8)	<.01
Tumor location						.02
Upper third, % (n)	15.5 (583)	15.3 (58)	23.6 (23)	33.3 (8)	33.3 (4)	
Middle third, %(n)	30.9 (1158)	37.7 (136)	30.2 (34)	33.3 (8)	25.0 (3)	
Lower third, %(n)	47.2 (1770)	41.5 (152)	43.4 (46)	33.3 (8)	33.3 (4)	
Postoperative stomach, %(n)	6.4 (240)	5.5 (20)	2.8 (3)	0 (0)	8.3 (1)	
General anesthesia, % (n)	3.4 (128)	10.7 (39)	33.0 (35)	41.7 (10)	58.3 (7)	
Procedure duration (minutes), mean ± SD	70.3 ± 42.6	121.2 ± 62.5	167.4 ± 65.9	245.9 ± 152.9	290.9 ± 108.1	<.01
Tumor diameter (mm), mean ± SD	13.3 ± 7.1	38.7 ± 5.4	62.5 ± 7.2	85.8 ± 6.8	121.3 ± 16.9	<.01
Tumor histopathology						<.01
Adenoma, % (n)	16.8 (631)	9.0 (33)	3.8 (4)	8.3 (2)	0 (0)	
Differentiated carcinoma, % (n)	71.7 (2688)	66.4 (243)	68.9 (73)	54.2 (13)	50.0 (6)	
Differentiated mixed type adenocarcinoma, % (n)	6.3 (238)	19.1 (70)	26.4 (28)	33.3 (8)	50.0 (6)	
Undifferentiated mixed type adenocarcinoma, % (n)	1.3 (49)	2.5 (9)	0.9 (1)	4.2 (1)	0 (0)	
Undifferentiated carcinoma, % (n)	3.9 (145)	3.0 (11)	0 (0)	0 (0)	0 (0)	
En bloc resection, % (n)	100.0 (3750)	100.0 (366)	100.0 (106)	100.0 (24)	100.0 (12)	.99
R0 resection, % (n)	96.1 (3632)	90.2 (330)	89.6 (95)	70.8 (17)	66.7 (8)	<.01
HM1 or HMX, % (n)	1.9 (73)	6.8 (25)	5.7 (6)	8.3 (2)	16.7 (2)	.50
VM1 or VMX, % (n)	1.4 (52)	3.8 (14)	6.6 (7)	20.1 (5)	25.0 (3)	<.01
Curative resection, % (n)	92.1 (3452)	61.2 (224)	63.2 (67)	45.8 (11)	8.3 (1)	<.01
Complication, % (n)						
Perforation	1.2 (45)	3.6 (13)	3.8 (4)	12.5 (3)	25.0 (3)	<.01
Postoperative bleeding	4.8 (179)	9.0 (33)	6.6 (7)	20.8 (5)	33.3 (4)	<.01
Delayed gastric emptying	0 (0)	0 (0)	5.7 (6)	4.2 (1)	0 (0)	<.01

ESD, endoscopic submucosal dissection; HM, horizontal margin; HMX, unable to assess cancer invasion of horizontal margin; VM, vertical margin; VMX; unable to assess cancer invasion of vertical margin.

**Table 2.** Noncurative Factors by Tumor Length

Tumor diameter (mm)	31-50	51-75	76-100	>101
Noncurative resection, n	38.8 (142)	36.8 (39)	54.2 (13)	91.7 (11)
SM invasion, % (n)	21.0 (77)	24.5 (26)	37.5 (9)	58.3 (7)
Deeper than SM2, % (n)	10.9 (40)	15.1 (16)	20.8 (5)	50.0 (6)
Undifferentiated-type	5.5 (20)	0.9 (1)	4.2 (1)	0 (0)
Lymphatic invasion, % (n)	8.2 (30)	11.3 (12)	25.0 (6)	16.7 (2)
Vascular invasion, % (n)	6.8 (25)	6.6 (7)	12.5 (3)	25.0 (3)
Ulceration (scar), % (n)	16.1 (59)	15.1 (16)	12.5 (3)	33.3 (4)
HM1 or HMX, % (n)	6.8 (25)	5.7 (6)	8.3 (2)	16.7 (2)
VM1 or VMX, % (n)	3.8 (14)	6.6 (7)	20.8 (5)	25.0 (3)
Additional treatment, n	142	39	13	11
Additional surgery	50.7 (72)	59.0 (23)	53.8 (7)	72.3 (8)
Follow-up	49.3 (70)	41.0 (16)	46.2 (6)	27.3 (3)
Primary disease deaths	0.3 (1)	0.9 (1)	0	0

HM, horizontal margin; HMX, unable to assess cancer invasion of horizontal margin; SM, submucosal; SM2, submucosal layer > 500µm from mucosal layer; VM, vertical margin; VMX, unable to assess cancer invasion of vertical margin.

**Table 3.** Cases of Gastric-Emptying Disorder due to Stenosis or Deformation after ESD

Case Number	Age (Years)	Sex	Location	Resection Size (mm)	Tumor Size (mm)	Range of Resected Mucosa/Circumference	Treatment	Additional Surgical Operation
1	84	Female	Antrum, GC	75 × 56	55 × 46	10/12	Dietary restrictions	None
2	79	Male	Antrum, LC	92 × 72	65 × 55	10/12	EBD twice	None
3	60	Male	Antrum, PW	72 × 70	60 × 51	11/12	Dietary restrictions	None
4	78	Male	Cardia, LC	115 × 75	75 × 36	12/12	EBD twice	None
5	88	Female	Cardia, AW	83 × 74	54 × 52	7/12	Dietary restrictions	None
6	86	Male	Antrum, GC	113 × 68	60 × 37	9/12	Dietary restrictions and nasogastric tube	None
7	81	Female	Antrum, LC	115 × 91	95 × 70	11/12	EBD 8 times	Distal gastrectomy

AW, anterior wall; EBD, endoscopic balloon dilation; ESD, endoscopic submucosal dissection; GC, greater curvature; LC, lesser curvature; PW, posterior wall.

## References

[1] Ono H Kondo H Gotoda T , et al. Endoscopic mucosal resection for treatment of early gastric cancer. Gut. 2001;48(2):225 229. (10.1136/gut.48.2.225) 11156645 PMC1728193

[2] Japanese Gastric Cancer Association. Japanese Gastric Cancer Treatment Guidelines 2018. 5th ed. Gastric Cancer 2021;24:1 21. 10.1007/s10120-020-01042-y PMC7790804 32060757

[3] Ichikawa T Kudo M Matsui S Okada M Kitano M . Endoscopic ultrasonography with three miniature probes of different frequency is an accurate diagnostic tool for endoscopic submucosal dissection. Hepatogastroenterology. 2007;54(73):325 328. 17419284

[4] Tsujii Y Kato M Inoue T , et al. Integrated diagnostic strategy for the invasion depth of early gastric cancer by conventional endoscopy and EUS. Gastrointest Endosc. 2015;82(3):452 459. (10.1016/j.gie.2015.01.022) 25841580

[5] Kuroki K Oka S Tanaka S , et al. Clinical significance of endoscopic ultrasonography in diagnosing invasion depth of early gastric cancer prior to endoscopic submucosal dissection. Gastric Cancer. 2021;24(1):145 155. (10.1007/s10120-020-01100-5) 32572791

[6] Hoteya S Iizuka T Kikuchi D , et al. Clinicopathological outcomes of patients with early gastric cancer after non-curative endoscopic submucosal dissection. Digestion. 2016;93(1):53 58. (10.1159/000441758) 26789628

[7] Kikuchi D Iizuka T Hoteya S , et al. Safety and efficacy of secondary endoscopic submucosal dissection for residual gastric carcinoma after primary endoscopic submucosal dissection. Digestion. 2012;86(4):288 293. (10.1159/000342114) 23051712

[8] Nomura K Hoteya S Kikuchi D Inoshita N Iizuka T . Utility of endoscopic submucosal dissection in the remnant stomach and clinical outcomes for different reconstruction methods. Digestion. 2019;100(4):254 261. (10.1159/000495346) 30485848

[9] Yamamoto H Kawata H Sunada K , et al. Successful en-bloc resection of large superficial tumors in the stomach and colon using sodium hyaluronate and small-caliber-tip transparent hood. Endoscopy. 2003;35(8):690 694. (10.1055/s-2003-41516) 12929067

[10] Japanese G astric C ancer S ociety. Guidelines for Diagnosis and Treatment of Carcinoma of the Stomach. Tokyo, Japan: Kanehara Shuppan; 2010.

[11] Oka S Tanaka S Kaneko I , et al. Advantage of endoscopic submucosal dissection compared with EMR for early gastric cancer. Gastrointest Endosc. 2006;64(6):877 883. (10.1016/j.gie.2006.03.932) 17140890

[12] Gotoda T Yanagisawa A Sasako M , et al. Incidence of lymph node metastasis from early gastric cancer: estimation with a large number of cases at two large centers. Gastric Cancer. 2000;3(4):219 225. (10.1007/pl00011720) 11984739

[13] Imagawa A Okada H Kawahara Y , et al. Endoscopic submucosal dissection for early gastric cancer: results and degrees of technical difficulty as well as success. Endoscopy. 2006;38(10):987 990. (10.1055/s-2006-944716) 17058162

[14] Yamagawa H Onishi T . A clinicopathological study of early gastric cancers with a diameter larger than five centimeters. Gan No Rinsho. 1989;35(10):1114 1118. 2550682

[15] Ishiguro S Kasugai T Terada N . Change of histological type of gastric carcinoma: from differentiated carcinoma to undifferentiated carcinoma. Stomach Intestine. 1996;31:1437 1443.

[16] Egashira Y Akutagawa H Umegaki E Higuchi K Hirose Y . The clinicopathologic feature of the early gastric cancer of differentiated and undifferentiated mixed type. Stomach Intestine. 2013;48:1553 1565.

[17] Coda S Oda I Gotoda T Yokoi C Kikuchi T Ono H . Risk factors for cardiac and pyloric stenosis after endoscopic submucosal dissection, and efficacy of endoscopic balloon dilation treatment. Endoscopy. 2009;41(5):421 426. (10.1055/s-0029-1214642) 19418396

[18] Sumiyoshi T Kondo H Minagawa T , et al. Risk factors and management for gastric stenosis after endoscopic submucosal dissection for gastric epithelial neoplasm. Gastric Cancer. 2017;20(4):690 698. (10.1007/s10120-016-0673-6) 27905029

